# Late Effects of Therapy in Childhood Acute Lymphoblastic Leukemia Survivors

**DOI:** 10.4274/tjh.galenos.2018.2018.0150

**Published:** 2019-02-07

**Authors:** Hande Kızılocak, Fatih Okcu

**Affiliations:** 1 Istanbul University-Cerrahpaşa Faculty of Medicine, Department of Pediatric Hematology and Oncology, İstanbul, Turkey; 2 Texas Children’s Hematology and Oncology Centers, Baylor College of Medicine, Department of Pediatrics, Division of Hematology and Oncology, Houston, TX, USA

**Keywords:** Acute lymphoblastic leukemia, Cancer survivorship, Late effects

## Abstract

Over the last 50 years, the survival rates in children with acute lymphoblastic leukemia (ALL) have increased remarkably. The optimal use of antileukemic agents in cooperative group protocols, central nervous system-directed treatment, improvements in supportive care, and recognition of biological, clinical, and treatment response characteristics that predict patients with a higher or a lower risk of treatment failure have improved 5-year event-free survival rates, reaching more than 85%, and 5-year overall survival rates, reaching more than 90%. Consequently, it has become increasingly important to characterize the occurrence of long-term late effects. ALL treatments have been associated with increased risks for adverse outcomes such as late mortality, secondary malignancies, and neurological, cardiac, endocrine, and social/psychological disorders. In recent decades, cooperative groups in Europe and in the United States have provided essential information about the long-term effects of ALL therapy, giving recommendations for screening as well as facilitating new approaches for reducing late-term morbidity and mortality. Current frontline protocols continue to examine ways to lower the intensity and amount of therapy to reduce late effects, whereas survivorship studies attempt to predict such adverse effects precisely and develop targeted prevention and treatment strategies.

## Abstract (tr)

Son 50 yıldaki gelişmeler ile akut lenfoblastik lösemili (ALL) çocuklardaki tedavi sonrası sağkalım oranında belirgin derecede artış saptandı. Antilösemik ilaçların akılcı kullanımı, santral sinir sistemini hedefleyen lokal tedaviler ve destek tedavisindeki gelişmeler ile 5 yıllık olaysız sağkalım %85’e ve 5 yıllık genel sağkalım %90’a ulaştı. Uzamış sağkalım süreleri, tedavinin uzun dönem yan etkilerine olan farkındalığı da arttırdı. Geç dönem mortalite, ikincil maligniteler, nörolojik, kardiyak, endokrin ve psikososyal bozukluklar ALL tedavisi sonrası sık görülen yan etkilerdir. Son yıllarda Avrupa ve Amerika Birleşik Devletleri’ndeki birçok grup ALL tedavisi sonrası uzun dönem yan etkiler konusunda çalışmalar yaptı, takip için ve uzun dönem mortalite ve morbiditeyi azaltmak için yeni yaklaşımlar önerdiler. Günümüzde kullanımda olan protokoller uzun dönem yan etkileri önlemek için tedavinin yoğunluğunu azaltmaya çalışırken, sağkalımla iligili çalışmalarda da yan etkilerin öngörülmesi ve gerekli önlem ve tedavilerin sağlanması konusu irdeleniyor.

## Introduction

In the last 50 years remarkable success in treating childhood acute lymphoblastic leukemia (ALL) has been achieved through modifications of chemotherapy and radiotherapy within cooperative group trials and improved supportive care [1]. As a result, it has become obvious that survivors of childhood ALL have, in the long term, increased risks of life-threatening severe late effects related to therapy (summarized in Table 1). In recent decades, cooperative groups in Europe and in the United States have prioritized development of treatment regimens aimed at reducing the risk for late effects without adversely impacting the cure rates. While traditional and molecular epidemiology studies are pursued to describe the growing spectrum of late effects seen in such survivors, development of interventions to prevent and treat late effects associated with significant mortality and morbidity has emerged as an important field of research. In this manuscript we present a comprehensive review of the epidemiology and burden of common late effects observed among childhood ALL survivors including secondary malignancies and neurological, cardiac, endocrine, and social/psychological disorders.

## Evolution of Acute Lymphoblastic Leukemia Therapy

The optimal use of antileukemic agents in cooperative group protocols, central nervous system (CNS)-directed treatment, improvements in supportive care, and recognition of biological, clinical, and treatment response characteristics able to predict patients at higher or lower risks of treatment failures have increased 5-year event-free survival (EFS) rates to above 85% and 5-year overall survival (OS) rates to above 90% [2], while few children survived 50 years ago [3]. 

After the initial single agent (aminopterin) and two-drug combinations (mercaptopurine and methotrexate) produced breakthrough “temporary remissions” in the 1940s and 1950s [4,5] Pinkel and colleagues developed a multiphase ALL treatment protocol in 1962 [6]. This included remission induction, CNS-directed therapy [including both cranial irradiation and intrathecal (IT) methotrexate], intensification (consolidation), and continuation treatment using a combination of 6-mercaptopurine and methotrexate. Such success inspired the development of similar clinical trials around the world, including two particularly important studies in the 1970s [7,8]. First the Berlin-Frankfurt-Munster group presented its “Protocol II” treatment, specifying a reinduction phase (i.e. the repetition of the initial remission induction therapy, which is now referred to as delayed intensification). Secondly, the Dana-Farber Cancer Center added weekly asparaginase at a high dose into its multiagent protocol. 

Simultaneously, in the 1970s, the use of prophylactic craniospinal radiation was the next major step in the evolution of treatment of ALL [9]. While only 2% of children with ALL have overt leukemia in the spinal fluid at diagnosis, approximately half will experience a CNS relapse if given systemic therapy alone. Craniospinal radiation led to several detrimental late effects including cognitive impairment, growth arrest, and panhypopituitarism in most of the patients [10,11]. To reduce these adverse outcomes first spine radiation was eliminated, followed by reductions in the cranial radiation dose from 24 to 18 Gy and then eventually to 12 Gy. Thus, in 1980s, cranial radiotherapy (CRT) became a standard component of successful multimodality therapy for treating and preventing CNS leukemia [12]. However, many children were still left with neurocognitive impairment that manifested as impaired processing speed, global intellectual function, and executive function. Ultimately a number of randomized studies showed that IT chemoprophylaxis with methotrexate or “triple” therapy with cytarabine, hydrocortisone, and methotrexate could replace CRT with no impact on outcome in the long term for most patients [13]. Only those patients with overt CNS disease at the time of diagnosis and some particular ALL subtypes [T-cell ALL with hyperleukocytosis or existence of overt CNS leukemia (i.e. CNS3 status)] with high risk for CNS failure are today treated with CRT. 

Hematopoietic stem cell transplantation (HSCT) has been applied with curative intent for cases of relapsed or high-risk ALL beginning in the late 1970s. HSCT recipients are exposed to chemotherapy and/or radiation before HSCT (for management of primary cancer), at HSCT, and after HSCT (for graft-versus-host disease and/or relapse of primary cancer), leading to early or late morbidity. Severe or life-threatening morbidity was reported in 40% of transplant cases, associated with premature mortality [14]. 

Ongoing recent investigational targeted therapy approaches include treatment with monoclonal antibodies such as blinatumomab and chimeric antigen receptor (CAR) T-cells. This area of research may, in the end, direct the development of therapies with high efficacy and lower risks of injury to normal tissues. At present, though, the long-term outcomes in children treated with these novel agents are unknown due to shorter follow-up times. 

As summarized above, the evolution of childhood ALL therapy has been a long and exciting process. While it represents a major success of cancer history with steady increases in survival over decades, a long list of associated late effects has been realized and will be described herein.

## Late Mortality

For childhood ALL patients 5-year OS estimates, including patients salvaged after relapse, are today >90% [3]. The Childhood Cancer Survivor Study (CCSS) cohort is a multicenter North American study examining the outcomes in childhood cancer patients who received their diagnoses between 1970 and 1999 and who survived for at least 5 years. Several reports from this cohort indicated that at 25 years from diagnosis, childhood ALL survivors have higher risks of early mortality beyond the 5-year mark [15,16,17] with a 13% cumulative incidence of death from any cause. The majority of deaths were due to leukemia recurrence (66%), yet compared to the sibling controls these patients were found to have a risk of dying from a subsequent cancer that was 15 times higher, a risk of dying from cardiac-related events that was 7 times higher, and a risk of dying from other medical conditions that was 2.6 times higher [15,16]. Mortality due to recurrence in ALL survivors was seen to decrease markedly as the follow-up was extended. Thus, at ≥10 years from the initial diagnosis, those ALL patients still surviving are seen to have low risks of recurrence and with rare exception are cured. There is a difference in OS between survivors treated with CRT (OS: 87.3%) and survivors who were not (OS: 96.1%) [18]. In a recent study that evaluated temporal trends in late mortality in a population of 34,033 patients in the CCSS cohort, reduction of mortality due to late effects related to treatment was reported in patients treated in the 1990s compared to earlier decades [19]. Reduced treatment intensity and increased use and accuracy of screening methods were associated with reduction in late mortality in patients with ALL.

## Secondary Malignancies

Secondary neoplasms (SNs) are among the most critical late effects of therapy for acute leukemia. Association of irradiation with the increased risk of SNs has been reported [20]. The chemotherapy agents that are most often associated with SN development are anthracyclines, etoposide, and alkylating agents [21]. ALL survivors are at increased risk for myelodysplastic syndrome (MDS), acute myeloid leukemia (AML), breast cancer, melanoma, CNS tumors, and non-Hodgkin’s lymphomas, as well as parotid and thyroid gland carcinomas [22,23]. 

The cumulative incidence of SNs for childhood ALL survivors has an overall range of 1%-11%, dependent on the treatment regimen used and the follow-up duration [22,24]. SNs are most commonly seen in skin (43%) and in the CNS (31%) [18]. Most secondary skin cancers are basal cell carcinomas, while 70% of the reported neoplasms of the CNS are meningiomas [15,18]. A retrospective study including 2169 childhood ALL patients who received treatment in 1962-1998 (median duration of follow-up was 18.7 years), the cumulative incidence of SNs was 4% overall at 15 years and was increased to 11% at 30 years [24]. Many of these reported late-developing SNs were “benign” neoplasms (basal cell carcinoma, n=14, 11.4%; and meningioma, n=16, 13%); upon exclusion of those diagnoses, cumulative incidence of SNs at 30 years was found to be lowered to 6%. The most common malignant SNs were 46 myeloid malignancies (AML, MDS), 3 lymphomas, 16 carcinomas excluding basal cell carcinoma, 6 sarcomas, and 22 brain tumors. Essig et al. [25] described the spectrum of late effects in 556 ALL survivors (median follow-up: 18.4 years) from the CCSS cohort who were 1-9.9 years old at diagnosis and who received a therapy similar to contemporary ALL protocols for patients with standard risk. Only six (1%) of them developed a subsequent malignancy (two melanomas, one astrocytoma, one large cell lymphoma, one leukemia, and one hepatocellular carcinoma). 

Radiation, either cranial or craniospinal, increases the risk of the development of secondary solid tumors. Nathan et al. [26] reported that such risk for SNs due to radiation is increased over time; they further found that neoplasms usually appeared at a minimum of 10 to 15 years following treatment. In a cohort containing 8831 pediatric patients who had been diagnosed with ALL and treated with the Children’s Cancer Group therapeutic protocols in 1983-1995, children receiving cranial radiation at 18 Gy were found to have lower risks of SNs compared to children receiving radiation at doses of 24 Gy [relative risk (RR): 1.5, with 95% confidence interval (CI) of 0.9-2.6; and RR: 3.9, with 95% CI of 1.4-11.2, respectively] [22]. The relative risk of secondary brain tumors following cranial radiation at a dose of 12 Gy, that being the dose that is used most often in the current protocols, has not yet been evaluated. 

As mentioned above, secondary AML can develop as well in ALL survivors, and it is seen at notably higher rates in patients who had been treated with more frequent or with higher doses of alkylating agents and epipodophyllotoxins [27]. As a result, the current regimens comprise comparatively lower cumulative doses of both cyclophosphamide and other alkylators. A recent study proposed the risk of secondary leukemia to be higher in those patients treated with higher initial doses of mercaptopurine in the maintenance phase (doses of 75 mg/m2/day compared to 50 mg/m2/day) [28]. It is still controversial whether patients having heterozygous or homozygous deficiencies of thiopurine methyltransferase face higher risks for SNs, but it is believed the risk may be partially dependent on the dose intensity of the mercaptopurine and/or its duration [29].

## Neurological Outcomes

### Cranial Radiotherapy

Survivors of childhood ALL face risks of neurocognitive late effects, which can include attention problems, impaired visual-motor function, decreased processing speed, and impaired working memory, which may affect their education achievement and quality of life (QOL) [30,31]. CNS-directed therapies, including CRT and/or IT chemotherapy, are established risk factors for impaired cognitive function, especially for younger patients [18]. In the initial 5-10 years following CRT, childhood ALL survivors have higher risks for neurocognitive skill deficits [2,32], and as they get older global brain injuries due to early CRT might decrease the cognitive reserve, putting these individuals at risk of memory impairments or early-onset dementia [3,33]. 

A study from the CCSS with 556 long-term ALL survivors who were CRT-naive and had been treated in the 1980s and 1990s reported that the survivors had lower overall functional statuses in spite of the fact that their neurocognitive deficits and mental health statuses were not different from those among their matched sibling donors [34]. Recent studies examining individuals treated with contemporary chemotherapy-only protocols uniformly reveal decreased performance in attention and processing speed in 20%-40% of survivors by the end of therapy [35,36,37].

### Methotrexate

Methotrexate (MTX) is the main agent associated with neurocognitive dysfunction in ALL survivors. A majority of published studies showed survivors receiving intravenous MTX at high does (>1 g/m^2^) to experience neurocognitive problems at higher rates than the ones who had been given MTX in low doses [38,39,40]. In another study, cumulative dosing of intravenous MTX heightened the risk for impairments in processing speed by increments of 3% for each 1 g/m^2^/dose [40]. IT MTX, and even in the absence of CRT, might be connected to changes in white matter, calcifications, cortical atrophy, and leukoencephalopathy, as well as seizures [41]. It is possible that IT cytosine arabinoside might increase the neurotoxicity of IT MTX [42]. Survivors of childhood ALL also face higher risks of fatigue and sleep disturbance [43,44], which are related to increases in neurocognitive impairments [45]. This might not only be related to side effects of drugs; it might also be due to longer hospitalization times. Folate pathway polymorphisms in the *MTHFR* 1298A>C and MS 2756A>G genes were reported in association with worse attention and processing speed in ALL survivors treated with contemporary protocols, suggesting a role for genetic predisposition [46,47].

### Corticosteroids

Corticosteroids remain a critical part of contemporary therapy for ALL and are most often administered as either prednisone or dexamethasone. While research among asthma patients suggested corticosteroids to be contributing to difficulties in cognitive functioning [48], earlier studies also showed that the particular steroid regimen chosen for treatment of ALL did not have a differential impact on neurocognitive functions in the long term [49]. The choice of corticosteroid, however, might have an effect on QOL in the long term. Toxicities that are both long-term and acute have been recorded with the usage of prednisone and dexamethasone, while findings suggest better CNS penetration of dexamethasone, which might possibly differentially influence QOL in the process of treatment and in the follow-up period.

### Vincristine

Vincristine, another critical tool in childhood ALL treatment, has been associated with dose-dependent peripheral neuropathy. In a study with 101 survivors, 16 (15.8%) of them were found to have a combination of electrophysiological and clinical neuropathy [50]. Those who were assigned to the intermediate- or high-risk arms of the chemotherapy protocol, which had cumulative vincristine doses of 36 mg/m^2^ or above, were found to have a ninefold increased risk for developing peripheral neuropathy in comparison to patients who had been treated according to the standard-risk arms and who received doses of 33 mg/m^2^ or lower. Peripheral neuropathy was related to lower QOL as well, but it was not found to be associated with worsened fine or gross motor functioning.

### Quality of Life (QOL)

QOL is a construct of multiple dimensions measuring well-being in a subjective manner. In a study that evaluated the contribution of neurocognitive dysfunction to QOL [51], 25% of the included survivors of ALL were found to be under the threshold for poor psychosocial QOL. Furthermore, 14% of them had poor physical QOL. The individuals with poor psychosocial QOL were much more likely to be males and were three times more likely to possess verbal deficits in their cognitive abilities and decreased skills for visual-motor integration. As in the general population, the family’s socioeconomic status plays a role in determining the QOL in childhood ALL patients. In particular, an association was found between low household income and poorer physical QOL, and also poorer emotional and social QOL. This implies that survivors belonging to socioeconomically disadvantaged households are especially susceptible to experience decreased QOL following the end of treatment. It is important that more attention be directed towards the determination of socioeconomic risk factors in order to assist at-risk patients and their families in overcoming barriers and accessing the resources they need to ensure the optimal psychosocial and physical functioning of their children.

## Cardiotoxicity

Childhood ALL survivors face risks of the development of late cardiotoxic effects of their treatments, especially after anthracycline therapy, including congestive heart failure (CHF), abnormalities of the heart valve, heart attacks, and heart epithelium inflammation [52,53]. Oxygen free radicals are produced by anthracyclines; they cause damage to cardiac myocytes, resulting in lost myofibrillar content and vacuolar degeneration, which in turn leads to fibrosis and myocardial necrosis. With the passage of time, thinning of the left ventricular wall occurs, which causes an increase in wall stress together with a decrease in myocardial contractility. Progressive cardiomyopathy might arise early on, during the first year of treatment, or it may occur later, with the diagnosis being given years after the end of the treatment [54,55]. 

Disease risk is dependent on the dose, CHF rates being ≤10% in those patients treated with cumulative doses of anthracyclines below 500 mg/m^2^ and as 36% for doses of >600 mg/m^2^ [56,57]. Furthermore, the risk of CHF related to the therapy is found to be altered by clinical factors such as age at time of treatment (<5 years of age), being female, preexisting heart disease, and simultaneous mediastinal irradiation [57,58,59,60]. In the CCSS cohort, cumulative 30-year incidences for CHF [4.1% (95% CI of 3.2%-5.0%)], myocardial infarction [1.3% (95% CI of 1.0%-1.7%)], valvular abnormalities [4.0% (95% CI of 3.1%-4.9%)], and pericardial disease [3.0% (95% CI of 2.1%-3.9%)] were increased in 10,367 survivors of childhood cancer who were then young adults in comparison to their siblings who served as control subjects [61]. As the anthracycline cardiotoxicity risk is dependent on the dose, practitioners have chosen to reduce the cumulative doses administered to patients with low risk in the last decade in order to decrease the occurrence of late cardiac dysfunction [52]. Where cumulative doses of >300 mg/m^2^ were applied, the risk for clinical heart failure at 20 years was estimated to be almost 10%, but where doses of <300 mg/m^2^ were applied, the predicted risk fell to 0.5% [62]. Worsening of cardiac function was also reported in a longitudinal follow-up following doses of >300 mg/m^2^. In a cross-sectionally designed study reporting on 138 survivors of childhood ALL who were then in adulthood and who had a median follow-up time of 23.4 years following diagnosis, LV functions were seen to be impaired in the patients (12%) who had received low cumulative doses of anthracycline (40-120 mg/m^2^) in their treatment [52], implying that there does not seem to be a certain safe dose when using anthracyclines [63,64]. Therefore, it is recommended to continue monitoring for any late cardiac effects in childhood survivors of ALL who experienced anthracycline exposure in order to prevent any advancement from asymptomatic cardiac dysfunction to clinical cases of CHF. 

Lastly, some evidence suggests that hypertension may be another cardiac late effect among ALL survivors. In a study that included 68 survivors who received treatment for childhood ALL between 1973 and 2000 and who were evaluated for the possibility of increased body mass index (BMI) and increased risk of high blood pressure, systolic and diastolic blood pressures were increased significantly compared to the general population (systolic blood pressure: mean z-score 0.736, p<0.001; diastolic blood pressure: mean z-score 0.409, p=<0.001; BMI: mean z-score 0.483, p<0.001). Female survivors, and especially those treated with CRT, had a greater risk of being overweight and obese, increasing the risk of cardiovascular morbidity later in life [65].

## Endocrine and Metabolic Outcomes

### Hormone Deficiencies

Deficiency of growth hormone (GH) is the most commonly seen endocrinopathy following radiation therapy in survivors of ALL due to direct injury to the hypothalamus [66,67]. It is dose-dependent and mostly seen at a dose of 24 Gy; however, it can also be seen at doses as low as 18 Gy, or at a single dose of 10 Gy applied during total body irradiation (TBI) [67,68]. Craniospinal radiation not only damages the hypothalamus but also directly affects skeletal growth, resulting in growth retardation [69,70]. Along with higher doses of CRT, the risk factors for GH deficiency also comprise younger age at the time of diagnosis and being female [67,68,69]. Additional central endocrinopathies, including central adrenal insufficiency, gonadotropin insufficiency, hyperprolactinemia, or central (or secondary) hypothyroidism, have also been associated with doses of 40 Gy [71], that dose being administered rarely in treating childhood ALL. The occurrence of primary hypothyroidism can also be seen following craniospinal or cranial radiation and TBI as a result of the thyroid gland’s direct exposure to radiation, even when doses of 10 Gy are applied, which is relatively low [72]. Another late effect of CRT when administered at doses of 18-24 Gy is precocious puberty; it is seen more commonly in females [73,74,75]. In two important cohort studies, the CCSS and the National Cancer Institute Children’s Cancer Group Leukemia Follow-up Study, a normal age of menarche was reported by 92% of female ALL survivors, in comparison with 97% and 96% of the studied controls, respectively [76,77]. 

Chemotherapy agents are not associated with endocrine late effects associated with hypothalamic pituitary axis injury [67]. In another CCSS report, compared to sibling controls, young adult ALL survivors were three times more likely to be diagnosed with a chronic endocrine disorder. The risk among survivors who did not relapse and who were not irradiated was comparable to that of the sibling control group [15].

### Obesity

There are conflicting reports on whether obesity is a true late effect in ALL survivors [78,79,80,81]. Most indicate that both adult and adolescent survivors present with BMIs higher than those seen in healthy controls, while others report no significant differences. Less attention has been paid to the loss of skeletal muscle mass, which leads to sarcopenia in cancer patients and is associated with the use of high-dose glucocorticosteroid therapy. In a cross-sectionally designed study of body composition in 75 long-term ALL survivors, sarcopenic obesity was present in 32 subjects (43%) [82]. Statistically significant and clinically important differences in overall health-related QOL between subjects with and without sarcopenic obesity were stated. A recent meta-analysis showed obesity to be prevalent in survivors still in childhood or those in early adolescence in comparison with reference populations [81]. Higher BMI was found among survivors to a significant extent, independently of sex, type of treatment, or duration from therapy completion to time of assessment. In another meta-analysis evaluating BMIs of adolescent and adult survivors of childhood ALL, patients were more likely by 12%-28% to be categorized as overweight or obese in comparison with the BMIs of the general population (p<0.001) [83]. Survivors of childhood cancer are seldom given recommendations about proper nutrition in the course of and subsequent to treatment. Such individuals need further resources for weight management, which could include advice regarding physical activity and support from dieticians, during the initial stages of their treatment (and particularly while steroids are being administered) and after the treatment has concluded. In a report evaluating chronic fatigue, the Fatigue Questionnaire was administered to 290 survivors of childhood lymphomas (n=139) and ALL (n=151) [84]. Among these long-term survivors of ALL, a subgroup was identified with persistent chronic fatigue. These individuals were found to have worse depressive symptoms, pain, and anxiety and they also engaged in physical activity less frequently, all of which can contribute to obesity.

### Infertility

Male survivors of childhood ALL in the long-term have an increased risk of experiencing infertility, gonadal dysfunction, and decreased semen quality due to the gonadotoxicity of some treatments, which include testicular irradiation and alkylating agents [85,86,87,88,89]. The relationship is dose-dependent for both patients treated with gonadal radiation at higher doses and with higher cumulative doses of alkylating agents, leading to higher risks [85]. CRT doses of 24 Gy or less do not lead to azoospermia in males [90]. 

In females, the CCSS reported that the RR for survivors of becoming pregnant at any time was 0.81 (95% CI: 0.73-0.90; p<0.001) in comparison with their female siblings. In multivariable models including survivors only, those treated with a hypothalamic/pituitary radiation doses of >30 Gy (RR, 0.61; 95% CI of 0.44-0.83) or ovarian/uterine radiation doses above 5 Gy were less apt to ever be pregnant (RR: 0.56 for 5 to 10 Gy, 95% CI of 0.37-0.85; RR: 0.18 for >10 Gy, 95% CI of 0.13-0.26) [91]. Among female survivors of cancer, the loss of primordial follicles at an accelerated rate due to damage to the gonads can cause premature ovarian failure, which can lead to gonadal failure, lowered ovarian reserve accompanied by lower antimüllerian hormone and higher gonadotrophin levels, and permanent infertility [92,93], making follow-up crucial. Additionally, the CCSS reported the occurrence of acute ovarian failure (AOF) in survivors at a rate of 6.3% [94]. The ovaries being exposed to radiation at high doses (particularly doses above 10 Gy), procarbazine, and alkylating agents were found to be important risk factors at older ages for AOF. In a retrospectively designed cohort study evaluating fertility (defined as ever being pregnant) by self-reporting in a population of 182 females and 170 sibling controls, the controls being female siblings of the survivors, deficits in fertility were recorded for survivors who had been treated with any does of CRT, and particularly those treated with it around menarche [95]. 

In a recent cohort (1987-2006) of French women who were survivors of childhood ALL, there was a negative correlation between fertility and alkylating agent use (p=0.01) and TBI (p=0.013) in univariate and multivariable analyses [96]. Fertility was also found with cumulative cyclophosphamide equivalent dose (p=0.001), fertility being seen to decrease for doses of ≥1 g/m^2^.

### Bone Toxicities

Changes in bone metabolism are considered as important adverse late effects, with pain, fractures, decrease of bone mineral density (BMD), and chronic impairment of bone function [97,98]. Treatment with high-dose MTX, mercaptopurine, glucocorticoids for 2-3 years, and CRT with low calcium intake, decreased physical activity, and obesity are some of the factors that lead to low BMD [99,100]. Furthermore, bone metabolism being impaired and bone mass being decreased in ALL patients who are newly diagnosed both imply that the disease directly affects BMD with leukemic infiltration of the bone marrow [101]. Survivors can recover lost bone mass during the post-treatment period, yet some will not reach their maximum BMD acquisition potential, presenting significant bone mass deficiency [102]. The first 2 years after the cessation of ALL therapy are the most critical for bone loss, with recovery taking place after that time [103]. 

In a single-institution study with 845 childhood ALL survivors (median age was 31 years), 5.7% displayed osteoporosis (BMD Z-score ≤-2) [104]. No correlation was found between cumulative doses of glucocorticoids and MTX and low BMD, but more exposure to prednisone was associated with decreased BMD in the female participants of the study. The strongest risk factor for a persistently low BMD (Z-score ≤-1) during young adulthood was high-dose (≥24 Gy) cranial or craniospinal radiation, associated with an approximate 2-fold increased risk compared to no cranial radiation exposure. 

In another single-center study with 101 patients [105], patients with Z-score values between -1.1 and -1.9 who were below 20 years of age had a higher cumulative risk of fractures (2%) and osteonecrosis (ON) (2%). The authors observed that lean mass was positively correlated with whole body and lumbar spine BMD values in patients under 20 years of age, confirming its importance for healthy development of bone mass. 

The incidence of symptomatic ON shows variation overall, with percentages ranging from 1% to 38% in the literature [106]. The association between ON and corticosteroids is described with two mechanisms. The first mechanism can be represented by direct vascular damage, which is characterized by processes of inflammation, and the second mechanism is gaseous microembolization following hepatotoxicity due to corticosteroids [107]. Vascular damage might justify the increased rate of ON of the femoral head and load of the humeral head [108]. The increased rate of necrosis at the femur head might be associated with the terminal type of vascularization at this level, as the collateral circulation does not compensate for alterations of the supply of blood. The same has been described for the humeral head, as it has vascularization and morphology that are similar to those of the femur. A retrospective analysis of 328 patients with ALL revealed only four (1.2%) cases of ON [109]. Between the diagnosis of ALL and ON, the median time was 12.5 months (range: 12-36 months). The femoral head was involved in all cases, in one case being associated with the scapula-humeral joint. ON is more often associated with dexamethasone in patients older than 10 years compared to prednisone [110].

## Social/Psychological Outcomes

Childhood ALL survivors have decreased educational attainment and also are less often married, employed, and parenting children than their siblings [15,111]. Cranial irradiation is the strongest predictor of these long-term effects [15,23]. Being young at the time of diagnosis and being female have been identified as the risk factors for worse future socioeconomic status [112]. In a registry-based study from Sweden involving 213 long-term childhood ALL survivors, female survivors were reported to possess higher risks of obtaining lower education levels than their male counterparts and population controls [113]. Younger age at the time of diagnosis was in association with lowered probability of marriage and children, along with lowered level of education with lower income after adjusting for the parents’ level of education. As was expected, therapy involving cranial irradiation was the main culprit for these associations, while steroids and IV/IT MTX were also statistically significant risk factors.

## Conclusion

More than 90% of childhood ALL survivors survive beyond 5 years after diagnosis, while many experience a long list of late effects from therapy, associated with late mortality, worse social and academic achievement, and worse QOL. Therapeutic exposures such as HSCT, CRT, and some chemotherapy agents put a significant number of ALL survivors at risk for multiple late effects including neurological and neurocognitive dysfunction, metabolic and endocrine abnormalities, bone toxicities, SNs, cardiac damage, and social/psychological adverse effects. Current trials are focused not only on maintaining high cure rates but also on further development of ALL treatments that minimize long-term adverse effects. The Children’s Oncology Group issued evidence-based guidelines available to any oncologist (http:/www.survivorshipguidelines.org/) for detection of late effects that may lead to remediation and improvement of overall health and QOL and prevention of late mortality. Thus, life-long ideal medical care in pediatric ALL survivors should include education on late effects and advice on adherence to periodic follow-up at a pediatric oncology center with an experienced survivorship team.

## Figures and Tables

**Table 1:**
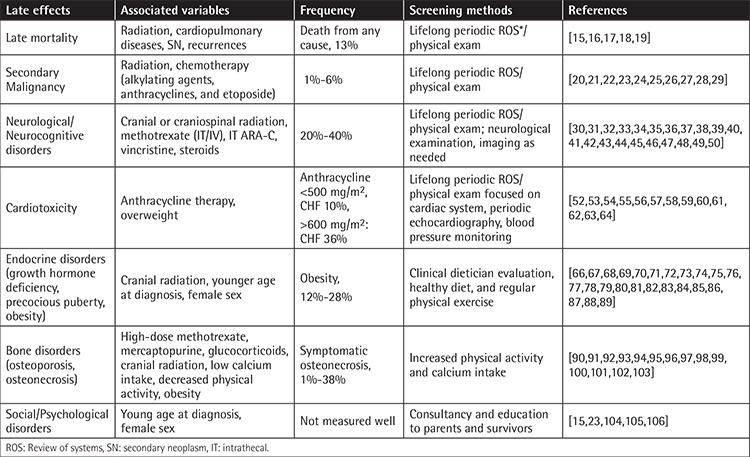
Summary of late effects in childhood acute lymphoblastic leukemia.

## References

[1] Carroll WL, Hunger SP (2016). Therapies on the horizon for childhood acute lymphoblastic leukemia. Curr Opin Pediatr.

[2] Pui CH, Campana D, Pei D, Bowman WP, Sandlund JT, Kaste SC, Ribeiro RC, Rubnitz JE, Raimondi SC, Onciu M, Coustan-Smith E, Kun LE, Jeha S, Cheng C, Howard SC, Simmons V, Bayles A, Metzger ML, Boyett JM, Leung W, Handgretinger R, Downing JR, Evans WE, Relling MV (2009). Treating childhood acute lymphoblastic leukemia without cranial irradiation. N Engl J Med.

[3] Hunger SP, Lu X, Devidas M, Camitta BM, Gaynon PS, Winick NJ, Reaman GH, Carroll WL (2012). Improved survival for children and adolescents with acute lymphoblastic leukemia between 1990 and 2005: a report from the Children’s Oncology Group. J Clin Oncol.

[4] Farber S, Diamond LK, Mercer RD (1948). Temporary remissions in acute leukemia in children produced by folic acid antagonist, 4-aminopteroyl-glutamic acid 374. N Engl J Med.

[5] Frei E, Freireich EJ, Gehan E, Pinkel D, Holland JF, Selawry O, Haurani F, Spurr CL, Hayes DM, James GW, Rothberg H, Sodee DB, Rundles RW, Schroeder LR, Hoogstraten B, Wolman IJ, Traggis DG, Cooper T, Gendel BR, Ebaugh F, Taylor R (1961). Studies of sequential and combination antimetabolite therapy in acute leukemia: 6-mercaptopurine and methotrexate. Blood.

[6] Pinkel D (1971). Five-year follow-up of “total therapy” of childhood lymphocytic leukemia. JAMA.

[7] Henze G, Langermann HJ, Brämswig J, Breu H, Gadner H, Schellong G, Welte K, Riehm H (1981). The BFM 76/79 acute lymphoblastic leukemia therapy study (author’s transl). Klin Padiatr.

[8] Sallan SE, Hitchcock-Bryan S, Gelber R, Cassady JR, Frei E 3rd, Nathan DG (1983). Influence of intensive asparaginase in the treatment of childhood non-T-cell acute lymphoblastic leukemia. Cancer Res.

[9] Carroll WL, Raetz EA (2012). Clinical and laboratory biology of childhood acute lymphoblastic leukemia. J Pediatr.

[10] Moleski M (2000). Neuropsychological, neuroanatomical, and neurophysiological consequences of CNS chemotherapy for acute lymphoblastic leukemia. Arch Clin Neuropsychol.

[11] Krull KR, Brinkman TM, Li C, Armstrong GT, Ness KK, Srivastava DK, Gurney JG, Kimberg C, Krasin MJ, Pui CH, Robison LL, Hudson MM (2013). Neurocognitive outcomes decades after treatment for childhood acute lymphoblastic leukemia: a report from the St Jude lifetime cohort study. J Clin Oncol.

[12] Follin C, Erfurth EM (2016). Long-term effect of cranial radiotherapy on pituitaryhypothalamus area in childhood acute lymphoblastic leukemia survivors. Curr Treat Options Oncol.

[13] Richards S, Pui CH, Gayon P; Childhood Acute Lymphoblastic Leukemia Collaborative Group (CALLCG) (2013). Systematic review and meta-analysis of randomized trials of central nervous system directed therapy for childhood acute lymphoblastic leukemia. Pediatr Blood Cancer.

[14] Bhatia S, Armenian SH, Landier W (2017). How I monitor long-term and late effects after blood or marrow transplantation. Blood.

[15] Mody R, Li S, Dover DC, Sallan S, Leisenring W, Oeffinger KC, Yasui Y, Robison LL, Neglia JP (2008). Twenty-five-year follow-up among survivors of childhood acute lymphoblastic leukemia: a report from the Childhood Cancer Survivor Study. Blood.

[16] Armstrong GT, Liu Q, Yasui Y, Neglia JP, Leisenring W, Robison LL, Mertens AC (2009). Late mortality among 5-year survivors of childhood cancer: a summary from the Childhood Cancer Survivor Study. J Clin Oncol.

[17] Mertens AC, Liu Q, Neglia JP, Wasilewski K, Leisenring W, Armstrong GT, Robison LL, Yasui Y (2008). Cause-specific late mortality among 5-year survivors of childhood cancer: the Childhood Cancer Survivor Study. J Natl Cancer Inst.

[18] Robison LL (2011). Late effects of acute lymphoblastic leukemia therapy in patients diagnosed at 0-20 years of age. Hematology Am Soc Hematol Educ Program.

[19] Armstrong GT, Chen Y, Yasui Y, Leisenring W, Gibson TM, Mertens AC, Stovall M, Oeffinger KC, Bhatia S, Krull KR, Nathan PC, Neglia JP, Green DM, Hudson MM, Robison LL (2016). Reduction in late mortality among 5-year survivors of childhood cancer. N Engl J Med.

[20] Fulbright JM, Raman S, McClellan WS, August KJ (2011). Late effects of childhood leukemia therapy. Curr Hematol Malig Rep.

[21] Borgmann A, Zinn C, Hartmann R, Herold R, Kaatsch P, Escherich G, Möricke A, Henze G, von Stackelberg A;, ALL-REZ BFM Study Group (2008). Secondary malignant neoplasms after intensive treatment of relapsed acute lymphoblastic leukaemia in childhood. Eur J Cancer.

[22] Bhatia S, Sather HN, Pabustan OB, Trigg ME, Gaynon PS, Robison LL (2002). Low incidence of second neoplasms among children diagnosed with acute lymphoblastic leukemia after 1983. Blood.

[23] Pui CH, Cheng C, Leung W, Rai SN, Rivera GK, Sandlund JT, Ribeiro RC, Relling MV, Kun LE, Evans WE, Hudson MM (2003). Extended follow-up of longterm survivors of childhood acute lymphoblastic leukemia. N Engl J Med.

[24] Hijiya N, Hudson MM, Lensing S, Zacher M, Onciu M, Behm FG, Razzouk BI, Ribeiro RC, Rubnitz JE, Sandlund JT, Rivera GK, Evans WE, Relling MV, Pui CH (2007). Cumulative incidence of secondary neoplasms as a first event after childhood acute lymphoblastic leukemia. JAMA.

[25] Essig S, Li Q, Chen Y, Hitzler J, Leisenring W, Greenberg M, Sklar C, Hudson MM, Armstrong GT, Krull KR, Neglia JP, Oeffinger KC, Robison LL, Kuehni CE, Yasui Y, Nathan PC (2014). Risk of late effects of treatment in children newly diagnosed with standard-risk acute lymphoblastic leukaemia: a report from the Childhood Cancer Survivor Study cohort. Lancet Oncol.

[26] Nathan PC, Wasilewski-Masker K, Janzen LA (2009). Long-term outcomes in survivors of childhood acute lymphoblastic leukemia. Hematol Oncol Clin North Am.

[27] Silverman LB (2014). Balancing cure and long-term risks in acute lymphoblastic leukemia. Hematology Am Soc Hematol Educ Program.

[28] Schmiegelow K, Levinsen MF, Attarbaschi A, Baruchel A, Devidas M, Escherich G, Gibson B, Heydrich C, Horibe K, Ishida Y, Liang DC, Locatelli F, Michel G, Pieters R, Piette C, Pui CH, Raimondi S, Silverman L, Stanulla M, Stark B, Winick N, Valsecchi MG (2013). Second malignant neoplasms after treatment of childhood acute lymphoblastic leukemia. J Clin Oncol.

[29] Schmiegelow K, Al-Modhwahi I, Andersen MK, Behrendtz M, Forestier E, Hasle H, Heyman M, Kristinsson J, Nersting J, Nygaard R, Svendsen AL, Vettenranta K, Weinshilboum R;, Nordic Society for Paediatric Haematology and Oncology (2009). Methotrexate/6-mercaptopurine maintenance therapy influences the risk of a second malignant neoplasm after childhood acute lymphoblastic leukemia: results from the NOPHO ALL-92 study. Blood.

[30] Krull KR, Okcu MF, Potter B, Jain N, Dreyer Z, Kamdar K, Brouwers P (2008). Screening for neurocognitive impairment in pediatric cancer long-term survivors. J Clin Oncol.

[31] Mulhern RK, Palmer SL (2003). Neurocognitive late effects in pediatric cancer. Curr Probl Cancer.

[32] Möricke A, Zimmermann M, Reiter A, Henze G, Schrauder A, Gadner H, Ludwig WD, Ritter J, Harbott J, Mann G, Klingebiel T, Zintl F, Niemeyer C, Kremens B, Niggli F, Niethammer D, Welte K, Stanulla M, Odenwald E, Riehm H, Schrappe M (2010). Long-term results of five consecutive trials in childhood acute lymphoblastic leukemia performed by the ALL-BFM study group from 1981 to 2000. Leukemia.

[33] Veerman AJ, Kamps WA, van den Berg H, van den Berg E, Bökkerink JP, Bruin MC, van den Heuvel-Eibrink MM, Korbijn CM, Korthof ET, van der Pal K, Stijnen T, van Weel Sipman MH, van Weerden JF, van Wering ER, van der Does-van den Berg A;, Dutch Childhood Oncology Group (2009). Dexamethasonebased therapy for childhood acute lymphoblastic leukaemia: results of the prospective Dutch Childhood Oncology Group (DCOG) protocol ALL-9 (1997-2004). Lancet Oncol.

[34] Essig S, Li Q, Chen Y, Hitzler J, Leisenring W, Greenberg M, Sklar C, Hudson MM, Armstrong GT, Krull KR, Neglia JP, Oeffinger KC, Robison LL, Kuehni CE, Yasui Y, Nathan PC (2014). Risk of late effects of treatment in children newly diagnosed with standard-risk acute lymphoblastic leukaemia: a report from the Childhood Cancer Survivor Study cohort. Lancet Oncol.

[35] Conklin HM, Krull KR, Reddick WE, Pei D, Cheng C, Pui CH (2012). Cognitive outcomes following contemporary treatment without cranial irradiation for childhood acute lymphoblastic leukemia. J Natl Cancer Inst.

[36] Krappmann P, Paulides M, Stöhr W, Ittner E, Plattig B, Nickel P, Lackner H, Schrappe M, Janka G, Beck JD, Langer T (2007). Almost normal cognitive function in patients during therapy for childhood acute lymphoblastic leukemia without cranial irradiation according to ALL-BFM 95 and COALL 06-97 protocols: results of an Austrian-German multicenter longitudinal study and implications for follow-up. Pediatr Hematol Oncol.

[37] Sands SA, Harel BT, Savone M, Kelly K, Vijayanathan V, Welch JG, Vrooman L, Silverman LB, Cole PD (2017). Feasibility of baseline neurocognitive assessment using Cogstate during the first month of therapy for childhood leukemia. Support Care Cancer.

[38] Aukema EJ, Caan MW, Oudhuis N, Majoie CB, Vos FM, Reneman L, Last BF, Grootenhuis MA, Schouten-van Meeteren AY (2009). White matter fractional anisotropy correlates with speed of processing and motor speed in young childhood cancer survivors. Int J Radiat Oncol Biol Phys.

[39] Krawczuk-Rybak M, Grabowska A, Protas PT, Muszynska-Roslan K, Holownia A, Braszko J (2012). Intellectual functioning of childhood leukemia survivors - Relation to Tau protein - A marker of white matter injury. Adv Med Sci.

[40] Krull KR, Brinkman TM, Li C, Armstrong GT, Ness KK, Srivastava DK, Gurney JG, Kimberg C, Krasin MJ, Pui CH, Robison LL, Hudson MM (2013). Neurocognitive outcomes decades after treatment for childhood acute lymphoblastic leukemia: a report from the St Jude lifetime cohort study. J Clin Oncol.

[41] Moleski M (2000). Neuropsychological, neuroanatomical, and neurophysiological consequences of CNS chemotherapy for acute lymphoblastic leukemia. Arch Clin Neuropsychol.

[42] Giralt J, Ortega JJ, Olive T, Verges R, Forio I, Salvador L (1992). Long-term neuropsychologic sequelae of childhood leukemia: comparisons of two CNS prophylactic regimens. Int J Radiat Oncol Biol Phys.

[43] Meeske KA, Siegel SE, Globe DR, Mack WJ, Bernstein L (2005). Prevalence and correlates of fatigue in long-term survivors of childhood leukemia. J Clin Oncol.

[44] Mulrooney DA, Ness KK, Neglia JP, Whitton JA, Green DM, Zeltzer LK, Robison LL, Mertens AC (2008). Fatigue and sleep disturbance in adult survivors of childhood cancer: a report from the childhood cancer survivor study (CCSS). Sleep.

[45] Clanton NR, Klosky JL, Li C, Jain N, Srivastava DK, Mulrooney D, Zeltzer L, Stovall M, Robison LL, Krull KR (2011). Fatigue, vitality, sleep, and neurocognitive functioning in adult survivors of childhood cancer: a report from the Childhood Cancer Survivor Study. Cancer.

[46] Kamdar KY, Krull KR, El-Zein RA, Brouwers P, Potter BS, Harris LL, Holm S, Dreyer Z, Scaglia F, Etzel CJ, Bondy M, Okcu MF (2011). Folate pathway polymorphisms predict deficits in attention and processing speed after childhood leukemia therapy. Pediatr Blood Cancer.

[47] Krull KR, Brouwers P, Jain N, Zhang L, Bomgaars L, Dreyer Z, Mahoney D, Bottomley S, Okcu MF (2008). Folate pathway genetic polymorphisms are related to attention disorders in childhood leukemia survivors. J Pediatr.

[48] Yeh TF, Lin YJ, Lin HC, Huang CC, Hsieh WS, Lin CH, Tsai CH (2004). Outcomes at school age after postnatal dexamethasone therapy for lung disease of prematurity. N Engl J Med.

[49] Waber DP, McCabe M, Sebree M, Forbes PW, Adams H, Alyman C, Sands SA, Robaey P, Romero I, Routhier MÈ, Girard JM, Sallan SE, Silverman LB (2013). Neuropsychological outcomes of a randomized trial of prednisone versus dexamethasone in acute lymphoblastic leukemia: findings from Dana- Farber Cancer Institute All Consortium Protocol 00-01. Pediatr Blood Cancer.

[50] Tay CG, Lee VWM, Ong LC, Goh KJ, Ariffin H, Fong CY (2017). Vincristine-induced peripheral neuropathy in survivors of childhood acute lymphoblastic leukaemia. Pediatr Blood Cancer.

[51] Kunin-Batson A, Kadan-Lottick N, Neglia JP (2014). The contribution of neurocognitive functioning to quality of life after childhood acute lymphoblastic leukemia. Psychooncology.

[52] Christiansen JR, Kanellopoulos A, Lund MB, Massey R, Dalen H, Kiserud CE, Ruud E, Aakhus S (2015). Impaired exercise capacity and left ventricular function in long-term adult survivors of childhood acute lymphoblastic leukemia. Pediatr Blood Cancer.

[53] Ness KK, Armenian SH, Kadan-Lottick N, Gurney JG (2011). Adverse effects of treatment in childhood acute lymphoblastic leukemia: general overview and implications for long-term cardiac health. Expert Rev Hematol.

[54] Adams MJ, Lipshultz SE (2005). Pathophysiology of anthracycline-and radiationassociated cardiomyopathies: implications for screening and prevention. Pediatr Blood Cancer.

[55] Berry GJ, Jorden M (2005). Pathology of radiation and anthracycline cardiotoxicity. Pediatr Blood Cancer.

[56] Grenier MA, Lipshultz SE (1998). Epidemiology of anthracycline cardiotoxicity in children and adults. Semin Oncol.

[57] Kremer LC, Van Dalen EC, Offringa M, Voute PA (2002). Frequency and risk factors of anthracycline induced clinical heart failure in children: a systematic review. Ann Oncol.

[58] Giantris A, Abdurrahman L, Hinkle A, Asselin B, Lipshultz SE (1998). Anthracyclineinduced cardiotoxicity in children and young adults. Crit Rev Oncol Hematol.

[59] Lipshultz SE, Lipsitz SR, Mone SM, Goorin AM, Sallan SE, Sanders SP, Orav EJ, Gelber RD, Colan SD (1995). Female sex and higher drug dose as risk factors for late cardiotoxic effects of doxorubicin therapy for childhood cancer. N Engl J Med.

[60] Pein F, Sakiroglu O, Dahan M, Lebidois J, Merlet P, Shamsaldin A, Villain E, de Vathaire F, Sidi D, Hartmann O (2004). Cardiac abnormalities 15 years adriamycin therapy in 229 childhood survivors of a solid tumour at the Institut Gustave Roussy. Br J Cancer.

[61] Mulrooney DA, Yeazel MW, Kawashima T, Mertens AC, Mitby P, Stovall M, Donaldson SS, Green DM, Sklar CA, Robison LL, Leisenring WM (2009). Cardiac outcomes in a cohort of adult survivors of childhood and adolescent cancer: retrospective analysis of the Childhood Cancer Survivor Study cohort. BMJ.

[62] van Dalen EC, van der Pal HJ, Kok WE, Caron HN, Kremer LC (2006). Clinical heart failure in a cohort of children treated with anthracyclines: a long-term follow-up study. Eur J Cancer.

[63] Rathe M, Carlsen NL, Oxhoj H, Nielsen G (2010). Long-term cardiac follow-up of children treated with anthracycline doses of 300 mg/m2 or less for acute lymphoblastic leukemia. Pediatr Blood Cancer.

[64] Lipshultz SE, Lipsitz SR, Sallan SE, Dalton VM, Mone SM, Gelber RD, Colan SD (2005). Chronic progressive cardiac dysfunction years after doxorubicin therapy for childhood acute lymphoblastic leukemia. J Clin Oncol.

[65] Veringa SJ, van Dulmen-den Broeder E, Kaspers GJ, Veening MA (2012). Blood pressure and body composition in long-term survivors of childhood acute lymphoblastic leukemia. Pediatr Blood Cancer.

[66] Vrooman LM, Stevenson KE, Supko JG, O’Brien J, Dahlberg SE, Asselin BL, Athale UH, Clavell LA, Kelly KM, Kutok JL, Laverdière C, Lipshultz SE, Michon B, Schorin M, Relling MV, Cohen HJ, Neuberg DS, Sallan SE, Silverman LB (2013). Postinduction dexamethasone and individualized dosing of Escherichia coli L-asparaginase each improve outcome of children and adolescents with newly diagnosed acute lymphoblastic leukemia: results from a randomized study--Dana-Farber Cancer Institute ALL Consortium Protocol 00-01. J Clin Oncol.

[67] Sklar CA, Constine LS (1995). Chronic neuroendocrinological sequelae of radiation therapy. Int J Radiat Oncol Biol Phys.

[68] Howard SC, Pui CH (2002). Endocrine complications in pediatric patients with acute lymphoblastic leukemia. Blood Rev.

[69] Chow EJ, Friedman DL, Yasui Y, Whitton JA, Stovall M, Robison LL, Sklar CA (2007). Decreased adult height in survivors of childhood acute lymphoblastic leukemia: a report from the childhood cancer survivor study. J Pediatr.

[70] Chemaitilly W, Sklar CA (2007). Endocrine complications of hematopoietic stem cell transplantation. Endocrinol Metab Clin North Am.

[71] Mills J, Bonner A, Francis K (2006). The development of constructivist grounded theory. International Journal of Qualitative Methods.

[72] DeGroot LJ (1993). Effects of irradiation on the thyroid gland. Endocrinol Metab Clin North Am.

[73] Leiper AD, Stanhope R, Kitching P, Chessells JM (1987). Precocious and premature puberty associated with treatment of acute lymphoblastic leukaemia. Arch Dis Child.

[74] Ogilvy-Stuart AL, Clayton PE, Shalet SM (1994). Cranial irradiation and early puberty. J Clin Endocrinol Metab.

[75] Quigley C, Cowell C, Jimenez M, Burger H, Kirk J, Bergin M, Stevens M, Simpson J, Silink M (1989). Normal or early development of puberty despite gonadal damage in children treated for acute lymphoblastic leukemia. N Engl J Med.

[76] Chow EJ, Friedman DL, Yasui Y, Whitton JA, Stovall M, Robison LL, Sklar CA (2008). Timing of menarche among survivors of childhood acute lymphoblastic leukemia: a report from the childhood cancer survivor study. Pediatr Blood Cancer.

[77] Mills JL, Fears TR, Robison LL, Nicholson HS, Sklar CA, Byrne J (1997). Menarche in a cohort of 188 long-term survivors of acute lymphoblastic leukemia. J Pediatr.

[78] Oeffinger KC, Mertens AC, Sklar CA, Yasui Y, Fears T, Stovall M, Vik TA, Inskip PD, Robison LL;, Childhood Cancer Survivor Study (2003). Obesity in adult survivors of childhood acute lymphoblastic leukemia: a report from the Childhood Cancer Survivor Study. J Clin Oncol.

[79] Shaw MP, Bath LE, Duff J, Kelnar CJ, Wallace WH (2000). Obesity in leukemia survivors: the familial contribution. Pediatr Hematol Oncol.

[80] Warner EL, Fluchel M, Wright J, Sweeney C, Boucher KM, Fraser A, Smith KR, Stroup AM, Kinney AY, Kirchhoff AC (2014). A population-based study of childhood cancer survivors’ body mass index. J Cancer Epidemiol.

[81] Zhang FF, Kelly MJ, Saltzman E, Must A, Roberts SB, Parsons SK (2014). Obesity in pediatric ALL survivors: a meta-analysis. Pediatrics.

[82] Marriott CJC, Beaumont LF, Farncombe TH, Cranston AN, Athale UH, Yakemchuk VN, Webber CE, Barr RD (2018). Body composition in long-term survivors of acute lymphoblastic leukemia diagnosed in childhood and adolescence: a focus on sarcopenic obesity. Cancer.

[83] Nam GE, Kaul S, Wu YP, Nelson RE, Wright J, Fluchel MN, Hacking CC, Kirchhoff AC (2015). A meta-analysis of body mass index of adolescent and adult survivors of pediatric acute lymphoblastic leukemia. J Cancer Surviv.

[84] Zeller B, Loge JH, Kanellopoulos A, Hamre H, Wyller VB, Ruud E (2014). Chronic fatigue in long-term survivors of childhood lymphomas and leukemia: persistence and associated clinical factors. J Pediatr Hematol Oncol.

[85] Green DM, Kawashima T, Stovall M, Leisenring W, Sklar CA, Mertens AC, Donaldson SS, Byrne J, Robison LL (2010). Fertility of male survivors of childhood cancer: a report from the Childhood Cancer Survivor Study. J Clin Oncol.

[86] Jahnukainen K, Heikkinen R, Henriksson M, Cooper TG, Puukko-Viertomies LR, Makitie O (2011). Semen quality and fertility in adult long-term survivors of childhood acute lymphoblastic leukemia. Fertil Steril.

[87] Greaves P, Sarker S, Chowdhury K, Johnson R, Matthews J, Matthews R, Smith M, Korszun A, Gribben JG, Lister TA (2014). Fertility and sexual function in long-term survivors of haematological malignancy: using patient-reported outcome measures to assess a neglected area of need in the late effects clinic. Br J Haematol.

[88] van Casteren NJ, van der Linden GH, Hakvoort-Cammel FG, Hahlen K, Dohle GR, van den Heuvel-Eibrink MM (2009). Effect of childhood cancer treatment on fertility markers in adult male long-term survivors. Pediatr Blood Cancer.

[89] Haavisto A, Henriksson M, Heikkinen R, Puukko-Viertomies LR, Jahnukainen K (2016). Sexual function in male long-term survivors of childhood acute lymphoblastic leukemia. Cancer.

[90] Green DM, Zhu L, Wang M, Chemaitilly W, Srivastava D, Kutteh WH, Ke RW, Sklar CA, Pui CH, Kun LE, Ribeiro RC, Robison LL, Hudson MM (2017). Effect of cranial irradiation on sperm concentration of adult survivors of childhood acute lymphoblastic leukemia: a report from the St. Jude Lifetime Cohort Study. Hum Reprod.

[91] Green DM, Kawashima T, Stovall M, Leisenring W, Sklar CA, Mertens AC, Donaldson SS, Byrne J, Robison LL (2009). Fertility of female survivors of childhood cancer: a report from the childhood cancer survivor study. J Clin Oncol.

[92] Gurgan T, Salman C, Demirol A (2008). Pregnancy and assisted reproduction techniques in men and women after cancer treatment. Placenta.

[93] El-Shalakany AH, Ali MS, Abdelmaksoud AA, Abd El-Ghany S, Hasan EA (2013). Ovarian function in female survivors of childhood malignancies. Pediatr Hematol Oncol.

[94] Green DM, Sklar CA, Boice JD Jr, Mulvihill JJ, Whitton JA, Stovall M, Yasui Y (2009). Ovarian failure and reproductive outcomes after childhood cancer treatment: results from the Childhood Cancer Survivor Study. J Clin Oncol.

[95] Byrne J, Fears TR, Mills JL, Zeltzer LK, Sklar C, Nicholson HS, Haupt R, Reaman GH, Meadows AT, Roçbison LL (2004). Fertility in women treated with cranial radiotherapy for childhood acute lymphoblastic leukemia. Pediatr Blood Cancer.

[96] Freycon F, Trombert-Paviot B, Casagranda L, Berlier P, Bertrand Y, Plantaz D, Stephan JL, Berger C;, Childhood Cancer Registry of the Rhône-Alpes Region Group (2015). Age at birth of first child and fecundity of women survivors of childhood acute lymphoblastic leukemia (1987-2007): a study of the Childhood Cancer Registry of the Rhône-Alpes Region in France (ARCERRA). Pediatr Hematol Oncol.

[97] Haddy TB, Mosher RB, Reaman GH (2001). Osteoporosis in survivors of acute lymphoblastic leukemia. Oncologist.

[98] Davies JH, Evans BA, Jenney ME, Gregory JW (2005). Skeletal morbidity in childhood acute lymphoblastic leukaemia. Clin Endocrinol (Oxf).

[99] Kohler JA, Moon RJ, Sands R, Doherty LJ, Taylor PA, Cooper C, Dennison EM, Davies JH (2012). Selective reduction in trabecular volumetric bone mineral density during treatment for childhood acute lymphoblastic leukemia. Bone.

[100] Watsky MA, Carbone LD, An Q, Cheng C, Lovorn EA, Hudson MM, Pui CH, Kaste SC (2014). Bone turnover in long-term survivors of childhood acute lymphoblastic leukemia. Pediatr Blood Cancer.

[101] Halton JM, Atkinson SA, Fraher L, Webber C, Gill GJ, Dawson S, Barr RD (1996). Altered mineral metabolism and bone mass in children during treatment for acute lymphoblastic leukemia. J Bone Mineral Res.

[102] Wasilewski-Masker K, Kaste SC, Hudson MM, Esiashvili N, Mattano LA, Meacham LR (2008). Bone mineral density deficits in survivors of childhood cancer: long-term follow-up guidelines and review of the literature. Pediatrics.

[103] Muszynska-Roslan K, Konstantynowicz J, Krawczuk-Rybak M, Protas P (2007). Body composition and bone mass in survivors of childhood cancer. Pediatr Blood Cancer.

[104] Gurney JG, Kaste SC, Liu W, Srivastava DK, Chemaitilly W, Ness KK, Lanctot JQ, Ojha RP, Nottage KA, Wilson CL, Li Z, Robison LL, Hudson MM (2014). Bone mineral density among long-term survivors of childhood acute lymphoblastic leukemia: results from the St. Jude Lifetime Cohort Study. Pediatr Blood Cancer.

[105] Molinari PCC, Lederman HM, Lee MLM, Caran EMM (2017). Assessment of the late effects on bones and on body composition of children and adolescents treated for acute lymphocytic leukemia according to Brazilian protocols. Rev Paul Pediatr.

[106] Aricò M, Boccalatte MF, Silvestri D, Barisone E, Messina C, Chiesa R, Santoro N, Tamaro P, Lippi A, Gallisai D, Basso G, De Rossi G;, Associazione Italiana di Ematologia ed Oncologia Pediatrica (2003). Osteonecrosis: an emerging complication of intensive chemotherapy for childhood acute lymphoblastic leukaemia. Haematologica.

[107] Felson DT, Anderson JJ (1987). A cross-study evaluation of association between steroid dose and bolus steroids and avascular necrosis of bone. Lancet.

[108] Sala A, Mattano LA Jr, Barr RD (2007). Osteonecrosis in children and adolescents with cancer: an adverse effect of systemic therapy. Eur J Cancer.

[109] Riccio I, Pota E, Marcarelli M, Affinita MC, Di Pinto D, Indolfi C, Del Regno N, Esposito M (2016). Osteonecrosis as a complication in pediatric patients with acute lymphoblastic leukemia. Pediatr Med Chir.

[110] Mattano LA, Sather HN, Trigg ME, Nachman JB (2000). Osteonecrosis as a complication of treating acute lymphoblastic leukemia in children: a report from the Children’s Cancer Group. J Clin Oncol.

[111] Lorenzi M, McMillan AJ, Siegel LS, Zumbo BD, Glickman V, Spinelli JJ, Goddard KJ, Pritchard SL, Rogers PC, McBride ML (2009). Educational outcomes among survivors of childhood cancer in British Columbia, Canada; Report of the childhood/adolescent/young adult cancer survivors CAYACS) program. Cancer.

[112] Harila-Saari AH, Lahteenmaki PM, Pukkala E, Kyyrönen P, Lanning M, Sankila R (2007). Scholastic achievements of childhood leukemia patients: a nationwide, register-based study. J Clin Oncol.

[113] Holmqvist AS, Wiebe T, Hjorth L, Lindgren A, Øra I, Moëll C (2010). Young age at diagnosis is a risk factor for negative late socio-economic effects after acute lymphoblastic leukemia in childhood. Pediatr Blood Cancer.

